# Omadacycline powder for research procured from unauthorized commercial vendors may be impure and/or unstable

**DOI:** 10.1128/aac.00638-24

**Published:** 2024-06-18

**Authors:** Alisa W. Serio, Daniel H. Deck, Kelly Wright, Kristen Manion

**Affiliations:** 1 Paratek Pharmaceuticals, Inc, King of Prussia, Pennsylvania, USA; Houston Methodist Hospital and Weill Cornell Medical College, Houston, Texas, USA

**Keywords:** omadacycline

## LETTER

Omadacycline tosylate is a third-generation tetracycline class antibiotic, approved by the U.S. Food and Drug Administration (FDA) in 2018 as a tablet and an intravenous (IV) formulation for the treatment of adults with community-acquired bacterial pneumonia and acute bacterial skin and skin structure infection (1). At the time of this writing, Paratek Pharmaceuticals, Inc. (Paratek) is the sole validated manufacturer of omadacycline tosylate. However, since its inception in the U.S. market in 2019, commercial vendors have offered researchers the ability to purchase omadacycline powder in various quantities (mg) and forms, e.g., tosylate, hydrochloride, and mesylate, with claims of >98% purity of the material on the respective Certificate of Analysis (COA). The product descriptions include claims specific to the omadacycline tosylate manufactured by Paratek, such as the mechanism of action, spectrum of activity, FDA-approved uses, clinical trials, and synonyms used early in the clinical development program (PTK0796) (1, 2).

Omadacycline tosylate from MedChemExpress has been used in at least 19 published studies (3), 6 of which are in ASM journals, 3 specifically in *Antimicrobial Agents and Chemotherap*y. Upon review of these 19 publications, omadacycline tosylate claims include, but are not limited to, *in vitro* potency, impact of resistance mechanisms on *in vitro* activity, and the propensity to select for mutations associated with resistance (3–10). As such, this analysis was performed to understand how the MedChemExpress powder performs when put through the validated, approved analytical methods used for the Paratek drug substance.

Paratek purchased omadacycline tosylate (lot 16053) from MedChemExpress and performed high-performance liquid chromatography and x-ray powder diffraction analysis according to the FDA-approved and validated assay/polymorphism procedures that are routinely performed to confirm the quality of Paratek’s FDA-approved omadacycline tosylate. The powder from MedChemExpress contained an assay of only ~53% (wt/wt) of omadacycline tosylate when compared to the Paratek omadacycline tosylate drug substance. In addition, it was confirmed that the MedChemExpress material is not the crystalline form of omadacycline tosylate drug substance but rather contains an amorphous diffraction pattern with no crystalline peaks detected. Therefore, the MedChemExpress material does not conform to the omadacycline tosylate form reference material and is a form of material that has very limited stability. The remaining material is composed primarily of known impurities [~40% (wt/wt)] that lack microbiological activity. The MedChemExpress material fails to meet regulatory approved and established standards of equivalence, >90.0% (wt/wt) assay, and does not conform to the stable omadacycline tosylate drug substance manufactured by Paratek (2). This analysis is limited in rebutting the MedChemExpress claim of 99.37% purity as the reference material supporting the claim is unknown.

We have identified at least 11 additional vendors who sell products named omadacycline in various forms also with claims of high quality (e.g., >98%) in their respective COAs, several of which have also been used in published studies. Given the lack of purity data of how these products compare to omadacycline tosylate manufactured by Paratek, their results should also be interpreted with caution.

In conclusion, when the Paratek-validated, approved analytical methods were utilized, the omadacycline tosylate powder from MedChemExpress failed to meet equivalence to Paratek’s FDA-approved omadacycline tosylate. As such these products should be considered distinct. We urge researchers as well as reviewers and editors to proceed with caution when evaluating research generated using omadacycline powders purchased from commercial suppliers unless there is appropriate validation when the true interest is in understanding attributes of omadacycline tosylate manufactured by Paratek.
